# Olive Mild Mosaic Virus Coat Protein and P6 Are Suppressors of RNA Silencing, and Their Silencing Confers Resistance against OMMV

**DOI:** 10.3390/v10080416

**Published:** 2018-08-09

**Authors:** Carla MR Varanda, Patrick Materatski, Maria Doroteia Campos, Maria Ivone E. Clara, Gustavo Nolasco, Maria do Rosário Félix

**Affiliations:** 1 ICAAM—Instituto de Ciências Agrárias e Ambientais Mediterrânicas, Instituto de Investigação e Formação Avançada, Universidade de Évora, Pólo da Mitra, Ap.94, 7006-554 Évora, Portugal; pmateratski@uevora.pt (P.M.); mdcc@uevora.pt (M.D.C.); 2 Departamento de Fitotecnia, ICAAM—Instituto de Ciências Agrárias e Ambientais Mediterrânicas, Escola de Ciências e Tecnologia, Universidade de Évora, Pólo da Mitra, Ap.94, 7006-554 Évora, Portugal; iclara@uevora.pt (M.I.E.C.); mrff@uevora.pt (M.d.R.F.); 3 MeditBio-Centro para os Recursos Biológicos e Alimentos Mediterrânicos, Faculdade de Ciências e Tecnologia, Universidade do Algarve, Campus de Gambelas, 8005-139 Faro, Portugal; gnolasco@ualg.pt

**Keywords:** RNA silencing, plant protection, resistance, plant viruses, viral suppressors

## Abstract

RNA silencing is an important defense mechanism in plants, yet several plant viruses encode proteins that suppress this mechanism. In this study, the genome of the *Olive mild mosaic virus* (OMMV) was screened for silencing suppressors. The full OMMV cDNA and 5 OMMV open reading frames (ORFs) were cloned into the Gateway binary vector pK7WG2, transformed into *Agrobacterium tumefaciens*, and agroinfiltrated into *N. benthamiana* 16C plants. *CP* and *p6* showed suppressor activity, with *CP* showing significantly higher activity than *p6*, yet activity that was lower than the full OMMV, suggesting a complementary action of *CP* and *p6*. These viral suppressors were then used to induce OMMV resistance in plants based on RNA silencing. Two hairpin constructs targeting each suppressor were agroinfiltrated in *N. benthamiana* plants, which were then inoculated with OMMV RNA. When silencing of both suppressors was achieved, a significant reduction in viral accumulation and symptom attenuation was observed as compared to those of the controls, as well as to when each construct was used alone, proving them to be effective against OMMV infection. This is the first time that a silencing suppressor was found in a necrovirus, and that two independent proteins act as silencing suppressors in a virus member of the *Tombusviridae* family.

## 1. Introduction

RNA silencing is a gene inactivation mechanism identified in most eukaryotes that is involved in several biological processes such as regulating endogenous gene expression, the maintenance of genome stability, and defense against viruses [1,2].

Amongst the several strategies plants have developed to counter virus infections, RNA silencing is one of the most important [1,3]. In antiviral RNA silencing, double-stranded RNAs (dsRNAs) are recognized as foreign and are processed into double-stranded viral short interfering RNAs (siRNAs) that are 21 nt to 22 nt long. Plant virus infections are associated with the accumulation of these virus-specific siRNAs. The cleavage is then accomplished by members of the Argonaute protein family (AGOs) [4] that recruit siRNA and associated proteins to form the RNA-induced silencing complex (RISC). This complex possesses ribonuclease activity and is guided by the single-stranded siRNAs to its target based on sequence complementarity, resulting in the binding and degradation of homologous RNA molecules [5,6]. Cleavage products of the target RNAs serve as a template of RNA-dependent RNA polymerases (RDR) to form dsRNAs, leading to secondary siRNA production [7], which can again initiate silencing in a self-sustained manner.

To counteract host RNA silencing defense, viruses have evolved several strategies. One such strategy involves viral proteins encoded in the genomes that suppress plant RNA silencing, termed viral suppressors of RNA silencing (VSRs) [8,9]. Most viruses studied have one VSR, and many VSRs have been identified [10]. These proteins are highly divergent, appearing to have evolved independently in the different viruses, and they may interfere in different stages of the RNA silencing pathways, either binding dsRNAs and inhibiting its processing into siRNAs, or sequestering viral siRNAs, preventing their incorporation into RISC or directly interfering with the recognition of viral RNA, dicing, and RISC assembly [6,11].

Viruses from different genera within the *Tombusviridae* family have different suppressors. The p19 protein of tombusviruses *Tomato bushy stunt virus* (TBSV) and *Cymbidium ringspot virus* (CymRSV), which is associated with long-distance movement, is one of the most studied viral suppressors [12,13,14]. The only function of the p14 protein of the aureusviruses, such as *Pothos latent virus*, seems to be to prevent silencing [15].

The CP of betacarmoviruses such as *Turnip crinkle virus*, as well as the movement proteins of some dianthoviruses, also act as silencing suppressors [16,17,18,19]. No silencing suppressors have been identified in the other genera in the family *Tombusviridae*, which would help to elucidate the evolutionary progression of viruses within this family.

The *Olive mild mosaic virus* (OMMV) is a member of the genus *Alphanecrovirus* within the *Tombusviridae* family, and is one of the most spread viruses in olive orchards [20,21,22]. Its genomic RNA is 3683 nt long with five open reading frames (ORFs), and the virus is likely to have resulted from recombination events between two other necroviruses *Olive latent virus 1* (OLV-1) and *Tobacco necrosis virus D* (TNV-D), based on the high amino acid identity with the RNA-dependent RNA polymerase of OLV-1 and of the CP of TNV-D [23]. ORF1 (p23), pre-readthrough, and ORF2 (p82), predicted as the RdRp, are involved in RNA replication; ORF3 (p8) and ORF 4 (p6) are predicted to be involved in virus movement; ORF5 (p29) is predicted as the CP, and it is involved in capsid assembly, systemic movement, and vector transmission [24].

In this study, OMMV-encoded proteins were examined to identify suppressors of RNA silencing and two were found, the CP and p6. OMMV silencing suppression ability seems to result from a coordinate and complementary action of both. In addition, resistance to OMMV in *N. benthamiana* was achieved using hpRNA constructs containing both *CP* and *p6*.

## 2. Materials and Methods

### 2.1. OMMV Silencing Suppressor Genes

#### 2.1.1. Generation of Constructs

Plasmid DNA containing the viral full-length of an OMMV clone (Accession number HQ651834.1) [25] was used for the amplification of OMMV *p23*, *p82*, *p8*, *p6* and *CP* ORFs as well as full-length OMMV. Each amplified sequence was cloned into pDONR^TM^221 (Invitrogen, Carlsbad, CA, USA) through a Gateway recombination reaction in accordance with the manufacturer’s instructions. The primers used in the reactions are listed in Table 1. Genes were transferred from pDONR^TM^221 to pK7WG2 binary vector [26] under the promotor CaMV *35S*, through LR recombination. Confirmation of the correct sequences was done by sequencing the constructs.

The *2b* suppressor gene of *Tomato aspermy virus* (TAV), which was used as positive control, and green fluorescent protein (GFP) (m-*gfp*5-ER) [27], which was used as a silencing inducer, were cloned as described previously [28].

Binary vectors were transformed into competent *Agrobacterium tumefaciens* strain GV3101/C58C1, carrying pMP90 Ti plasmid, which confers resistance to gentamycin. *A. tumefaciens* cultures were placed individually in 15 mL of Luria-Bertani (LB) medium supplemented with gentamycin, spectinomycin, and rifampicin at 50 μg mL^−1^ each, 10 mM of MES and 20 μM of acetosyringone. Cultures were then grown at 28 °C and 200 rpm until reaching an OD_600_ of 0.5. Cells were sedimented, re-suspended, in a 3-mL volume containing 10 mM of MgCl_2_ (pH 5.6), 10 mM of MES and 100 μM of acetosyringone, and kept in the absence of light at room temperature for 1 h before infiltration [29].

#### 2.1.2. Agrobacterium Co-Infiltration Assays and GFP Imaging

Silencing suppressor assays were based on the previously described system [29]. Briefly, when leaves of *GFP* transgenic 16C line of *N. benthamiana* plants, constitutively expressing the *GFP* gene, are agroinfiltrated with *A. tumefaciens* carrying a *GFP* construct, the green fluorescent signal disappears under UV light due to *GFP* silencing. However, if the *GFP* construct is co-infiltrated with a silencing suppressor, the fluorescence does not disappear, and may even become more intense due to the inhibition of gene silencing caused by the suppressor.

For transient expression assays, Agrobacterium cultures carrying each construct were infiltrated into leaves of four-week-old *N. benthamiana* 16C line plants, gently provided by David Baulcombe (University of Cambridge, Cambridge, UK). Single and co-infiltration assays were performed using a 5-mL needleless syringe. Single infiltration consisted of using a 15-mL suspension of *A. tumefaciens* carrying pK_*GFP*. For co-infiltration assays, Agrobacterium cultures containing each construct individually, including Tav-*2b*, and *GFP* were mixed in 1:1 *v*/*v* ratio before agroinfiltration, centrifuged, and resuspended in a final volume of 15 mL.

Three leaves per plant and 10 plants (biological replicates) per each construct were infiltrated. Plants were observed during 12 days post-infiltration (dpi). Each experiment was repeated three times.

The GFP fluorescence of infiltrated leaves and whole plants was examined using a long-wavelength UV lamp (UVPBlak-Ray B-100AP, ThermoFisherScientific, Waltham, MA, USA and photographed with a digital camera (Sony α100 DSLR-A100K, Sony, Tokyo, Japan).

#### 2.1.3. RNA Extraction and Real-Time RT-PCR

Total RNA was extracted from the leaves of three randomly selected agroinfiltrated plants (biological replicates) at 3 dpi, 5 dpi, and 10 dpi, using RNeasy Plant Mini Kit (Qiagen, Hilden, Germany) in accordance with the manufacturer’s instructions. The quality and concentration of all of the RNA preparations were determined by using a Nanodrop 2000c spectrophotometer (Thermo Scientific, Waltham, MA, USA).

For reverse transcription, 1 μg of total RNA was used in a 20-μL reaction using Maxima^®^ First Strand cDNA Synthesis Kit for RT-qPCR (Thermo Scientific) in accordance with the manufacturer’s instructions.

Primers were designed using Primer Express 3.0 software for real-time PCR (Applied Biosystems, Foster City, CA, USA) using the default parameters for the software (Table 2). Quantitative assays of *GFP* mRNA were performed by real-time RT-PCR (RT-qPCR), carried out on a 7500 Real Time PCR System (Applied Biosystems).

A mRNA *GFP* 147-bp fragment, OMMV ORFs (*p23*, *p82*, *p8*, *p6*, *CP*), as well as full-length OMMV, were amplified. The protein endogenous control genes phosphatase 2A (*PP2*) and F-box protein (*F-box*) were used as internal standards.

RT-qPCRs were carried out with 12.5 μL of 2× SYBR Green PCR Master Mix, 0.3 μM of each primer, and 12.5 ng of cDNA per sample, prepared in 96-well plates and run for 40 amplification cycles comprising a 15-s denaturation at 95 °C, followed by a 1 min at a 60 °C step. A negative control with no template and three technical replicates were considered.

Cycle threshold (C_T_) values were determined using the fit-point method and the Applied Biosystems 7500 software with a fluorescence threshold arbitrarily set to 0.1. The relative level of *GFP* mRNA was determined using the amount of *GFP* mRNA from 16C non-inoculated plants as the reference level. At the end of the qPCR, melt curve analysis was conducted to validate the specificity of the primers. A standard curve for each gene was automatically generated by the instrument software (Applied Biosystems) for relative expression level estimation, and data was normalized by *PP2* and *F-box* (reference genes). To exclude the possibility of weak or no silencing suppression due to low levels of the respective protein expression in infiltrated leaves, quantitative assays of the mRNA of each the proteins were performed by RT-qPCR, as described previously for *GFP*.

#### 2.1.4. Data Analysis

Univariate and multivariate analyses were performed using the PRIMER v6 software [31] with the permutational analysis of variance (PERMANOVA) add-on package [32], to detect significant differences (*p* < 0.05) in the relative *GFP* mRNA levels between: “ORFs” *p23*, *p82*, *p8*, *p6*, *CP*, OMMV, *GFP* and Tav-*2b*, and “Time” day 3, day 5, and day 10. A two-way PERMANOVA was applied to test the null hypotheses that no significant differences existed between “ORFs” and “Time”. PERMANOVA analyses were carried out with the following two-factor design: ORFs *p23*, *p82*, *p8*, *p6*, *CP*, OMMV, *GFP*, and Tav-*2b* (eight levels, fixed); and Time, day 3, day 5 and day 10 (three levels, fixed). The data were square root transformed in order to scale down the importance of the high values of relative *GFP* mRNA levels. The PERMANOVA analysis was conducted on a Bray–Curtis similarity matrix [33]. If the number of permutations was lower than 150, the Monte Carlo permutation p was used. Whenever significant interaction effects were detected, these were examined using a posteriori pairwise comparisons, using 9999 permutations under a reduced model.

### 2.2. OMMV Resistance Challenge

A full-length cDNA of OMMV (Accession number HQ651834.1) was used for in vitro transcription using RiboMax^TM^ Large Scale RNA Production System-T7 (Promega, Madison, WI, USA). Following transcription, DNA templates were removed by digestion with DNase, and transcripts were purified by extraction with phenol:chloroform (5:1) acid equilibrated (pH 4.7) (Sigma, St. Louis, MO, USA) and ethanol precipitated.

Synthesized RNA was mechanically inoculated onto six to eight leaf stage *N. benthamiana* plants maintained in a growth chamber at 23 °C with a 16-h photoperiod, for viral propagation. OMMV-infected leaves were then ground in cold 0.1 M of sodium phosphate buffer, pH 6.0 (1:3 *w*/*v*), which was then filtered, clarified in the presence of organic solvents, concentrated by differential centrifugation, and further purified by ultracentrifugation through sucrose density gradients [34]. The concentration of viral preparations was determined by using a Nanodrop 2000c spectrophotometer (Thermo Scientific) prior to inoculation.

Hairpin constructs of *CP* and *p6* were constructed based on the *CP* without the first 141 nt to exclude CP-mediated resistance events (using OMMVCP-141attB1: 5′ AAAAAGCAGGCTATCCTAGATCTTCTGGGCTAAGC and OMMVCPattB2) and on the full *p6* (using OMMVp6attB1 and OMMVp6attB2), respectively. hpRNA-*CP* and hpRNA-*p6* constructs were obtained through LR recombination from each pDONR^TM^221-*CP* and pDONR^TM^221-*p6*, as described previously, to pHELLSGATE12 [35], placed in sense and antisense directions to produce self-complementary dsRNAs. Confirmation of the correct sequences was done by sequencing of the constructs after linearization with *Cla*I, which cleaves within the intron.

15 μg of purified OMMV were inoculated onto two fully expanded carborundum-dusted leaves of four-week-old *N. benthamiana* 16C line plants three days after infiltration with Agrobacterium cultures carrying pHELLSGATE12-*CP* and pHELLSGATE12-*p6*, as described for transient expression assays. Inoculated plants were grown in the conditions mentioned above. The presence of each construct was confirmed by RT-qPCR at 3dpi, as shown previously.

Ten plants (biological replicates) were infiltrated with pHELLSGATE12-*CP*, 10 were infiltrated with pHELLSGATE12-*p6*, 10 were infiltrated with both constructs, and 10 were infiltrated with the empty vector to be used as negative control. All 40 plants were mechanically inoculated with OMMV. Experiments were repeated two times.

Plants were monitored daily for 30 days for symptom development. A four-grade disease scale was adopted to describe OMMV symptoms along time: 0, no symptoms; 1, mild chlorotic mosaic; 2, intense chlorotic mosaic; 3, necrotic mosaic; 4, pronounced leaf necrosis; and deformation. Disease severity was evaluated at two dpi, five dpi, 10 dpi, and 16 dpi for each batch of plants infiltrated with the different constructs, as follows: Disease Severity Index (DSI) = (SUM of all disease ratings/(Total number of ratings × Maximum disease grade)) × 100.

Total RNAs were extracted from upper non-inoculated leaves from three randomly selected plants (biological replicates), at five dpi and 16 dpi. Virus accumulation was determined by quantitative real time PCR analysis using OMMVp23 primers (Table 2), as described above.

Univariate and multivariate analyses were performed as described above, using the PRIMER software to detect significant differences (*p* < 0.05) in the virus accumulation between; “ORFs” *p6*, *CP*, and *p6* + *CP*, and “Time” five dpi and 16 dpi. A two-way PERMANOVA was applied to test the null hypotheses that no significant differences existed between “ORFs” and “Time”. PERMANOVA analyses were carried out as previously, with the following two-factor design: ORFs *p6*, *CP*, and *p6* + *CP* (three levels, fixed); and Time five dpi and 16 dpi (two levels, fixed). The data were square-root transformed in order to scale down the importance of the high values of virus accumulation.

## 3. Results

### 3.1. Determination of OMMV Silencing Suppressors

To identify potential OMMV RNA silencing suppressors, all of the proteins encoded by the genome were tested for their ability to suppress silencing by using a *GFP* reporter gene in plant tissues. Individual OMMV ORFs were cloned into a binary vector (pK7WG2) driven by the CaMV *35S* promoter in *A. tumefaciens*. For comparison, a full-length cDNA clone of OMMV that generates a full infection in *N. benthamiana* was also cloned. Transient expression assays were performed on transgenic 16C *N. benthamiana* plants by co-infiltrations of each clone plus pK_*GFP*, a construct that expresses a transcript homologous to the transgene of 16C plants.

**Figure 1:**
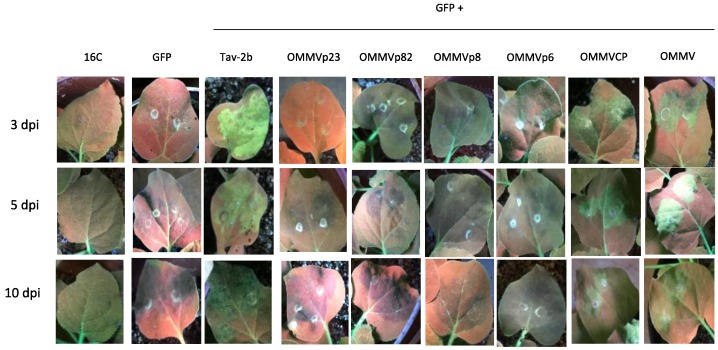
Visual observation of leaves from transgenic *N. benthamiana* 16C plants under UV light at three, five, and 10 days post-infiltration (dpi). Non-infiltrated (16C), infiltrated with *Agrobacterium tumefaciens* harboring pK-*GFP* alone (GFP), and co-infiltrated with pK-*GFP* plus: Tav-*2b*, OMMV*p23*, OMMV*p82*, OMMV*p8*, OMMV*p6*, OMMV*CP*, and OMMV.

Infiltrated *N. benthamiana* leaf patches showed GFP fluorescence under UV light at two dpi in all of the samples (data not shown). Leaves infiltrated with the silencing inducer pK_*GFP* showed a weak GFP fluorescence at three dpi (Figure 1, GFP 3dpi) and at five dpi, fluorescence disappeared (Figure 1, GFP 5 dpi) and silencing began, as seen by the development of a red color under UV light on the infiltrated region.

In the positive control, leaves co-infiltrated with pk_*GFP* and the strong suppressor Tav-*2b* presented bright fluorescence at three dpi (Figure 1, Tav-*2b* 3 dpi), and fluorescence was maintained for the next seven days, although it was less intense (Figure 1, Tav-*2b* 10 dpi). 

In the co-expression of OMMV*p23*, OMMV*p82*, and OMMV*p8*, at three dpi, five dpi, and 10 dpi, no fluorescence was observed under UV light, and silencing began at five dpi and was maintained at 10 dpi.

At three dpi, five dpi, and 10 dpi, OMMV*p6*, OMMV*CP*, and OMMV were able to suppress *GFP* silencing, as seen by the green fluorescence under UV light.

OMMV*CP* and OMMV showed the most intense suppressor activity at five dpi, whereas OMMV*p6* showed the most intense suppressor activity at three dpi. However, OMMV*p6* green fluorescence showed lower intensity at all times when compared to OMMV*CP* and OMMV, and was almost undetectable at 10 dpi.

GFP fluorescence in the presence of pk_OMMV was reproducibly stronger than with each of the viral genes, and was similar to the levels observed for Tav-*2b*. However, in contrast to Tav-*2b*, where higher levels were observed at three dpi, a brighter fluorescence was observed at five dpi.

Monitoring of upper non-infiltrated leaves showed systemic silencing at 15 dpi in all of the samples, suggesting that OMMV*p6* and OMMV*CP* possess suppressor local RNA silencing activity, but cannot suppress systemic silencing.

RT-qPCR showed that all of the samples presented C_T_ values within the linear calibration curves. Two reference genes (*PP2* and *F-box*) were used to normalize the expression of target genes. The amplification efficiency and correlation coefficient (R^2^) of their calibration curves were 107.25% and 0.9943% for *PP2*, and 99.62% and 0.9978% for *F-box*.

**Figure 2:**
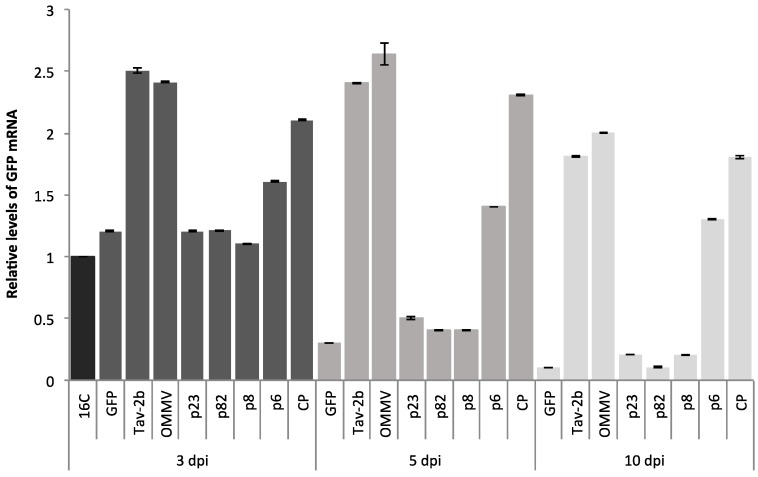
Mean relative levels of *GFP* mRNA ± standard error (SE) obtained from three infiltrated 16C *N. benthamiana* plants (biological replicates) at three dpi, five dpi, and 10 dpi, determined by RT-qPCR and normalized by the levels of phosphatase 2A (*PP2*) and F-box protein (*F-box*) reference genes. 16C: 16C non-inoculated plants; GFP: single infiltration with pk_*GFP*; Tav-*2b*, OMMV*p23*, OMMV*p82*, OMMV*p8*, OMMV*p6*, OMMV*CP*, and OMMV: co-infiltration of pk_*GFP* with the corresponding constructs. The *GFP* transgene level in non-infiltrated *N. benthamiana* 16C plants was used as a standard and given a value of 1.

PERMANOVA analysis revealed significant differences in the factors “ORFs” and “Time” (*p* < 0.0001). In addition, a significant interaction occurred between the two factors (“ORFs” and “Time”) (*p* < 0.0001). As expected by visual observations, RT-qPCR at three dpi showed the highest values (mean ± SE) of relative *GFP* mRNA levels; 2.50 ± 0.01 in Tav-*2b* gene, followed by 2.41 ± 0.01 in OMMV, 2.10 ± 0.005 in *CP*, and 1.60 ± 0.004 in *p6* (Figure 2). Individual pairwise comparisons at three dpi revealed a high variability of relative *GFP* mRNA levels with significant differences between most ORFs (*p* < 0.05) (Table 3). Individual pairwise comparisons showed no significant differences between: *p GFP* versus *p23* > 0.9153, *p p23* versus *p82* > 0.6178 and *p p23* versus *p82* > 0.088.

At five dpi, RT-qPCR showed a great reduction in *GFP* mRNA levels in the presence of GFP alone or in the presence of OMMV*p23*, OMMV*p82*, and OMMV*p8*. Consistent with visual observations, at five dpi, the highest values (mean ± SE) of relative *GFP* mRNA levels were 2.64 ± 0.09 in OMMV (1.6-fold greater than non-infiltrated 16C at five dpi), followed by 2.40 ± 0.005 in Tav-*2b*, 2.30 ± 0.004 in *CP*, and 1.40 ± 0.002 in *p6* (Figure 2). Individual pairwise comparisons at five dpi revealed a high variability of relative *GFP* mRNA levels with significant differences between most of the ORFs (*p* < 0.05) (Table 3). Individual pairwise comparisons revealed no significant differences between: *p* Tav-*2b* versus OMMV > 0.0518 and *p p82* versus *p8* > 0.9977.

At 10 dpi, all of the *GFP* levels decreased, the highest values (mean ± SE) of relative *GFP* mRNA levels were 2.00 ± 0.005 in OMMV, followed by 1.81 ± 0.005 in Tav-*2b*, 1.80 ± 0.01 in *CP*, and 1.30 ± 0.003 in *p6* (Figure 2) (from 0.3 in OMMV*p6* to onefold greater than non-infiltrated 16C plants in OMMV). Individual pairwise comparisons at 10 dpi revealed a high variability of relative *GFP* mRNA levels with significant differences between most of the ORFs (*p* < 0.05) (Table 3). Individual pairwise comparisons revealed no significant differences between: *p GFP* versus *p82* > 0.3574, *p p23* versus *p82* > 0.6807, *p p23* versus *p8* > 0.6569, *p p82* versus *p8* > 0.6799 and *p* Tav-*2b* versus *CP* > 0.8368.

Plants co-infiltrated with pk_*GFP* and OMMV*CP* and pK_*GFP* and OMMV maintained the greenish patch for 11 and 14 days, respectively.

At three dpi and five dpi, expression in leaves co-infiltrated with pk_*GFP* and each OMMV*p23*, OMMV*p82*, OMMV*p8*, and OMMV*p6*, was similar to that of the strong silencing suppressor OMMV*CP*. This result confirms that proteins were being expressed, and demonstrated that weak or no silencing suppression was not due to low protein expression.

### 3.2. OMMV Resistance Challenge

The presence of each construct was confirmed by RT-qPCR at three dpi in plants infiltrated with Agrobacterium cultures carrying the constructs.

**Figure 3:**
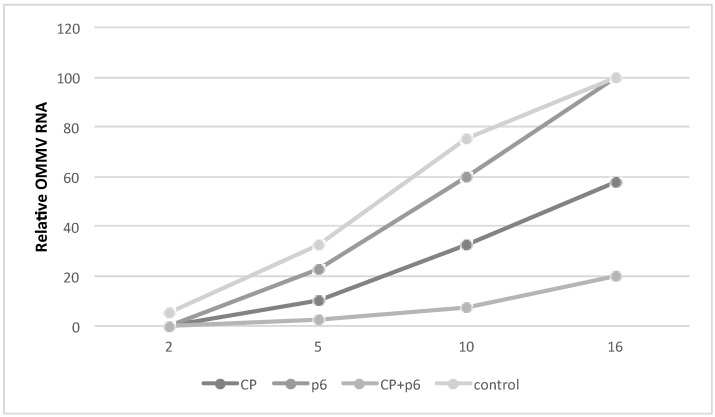
Disease severity of plants (10 biological replicates) expressing OMMV *CP*, OMMV *p6*, OMMV*CP* + *p6*, and negative control at two dpi, five dpi, 10 dpi, and 16 days after inoculation of OMMV.

The appearance of disease symptoms was monitored at two dpi, five dpi, 10 dpi, and 16 dpi and recorded as a disease index scale. As shown in Figure 3, at two dpi, plants expressing OMMV *CP*, OMMV *p6*, and both OMMV *CP* and *p6* did not show any symptoms, whereas two plants in negative control showed mild symptoms (DSI, 5%). At five dpi, plants from all groups showed symptoms, but only a few plants in the negative control group reached the disease index scale of 2, presenting the highest DSI (32.5%), followed by plants expressing *p6* alone (22.5%). At 10 dpi and 16 dpi, control plants showed severe symptoms (DSI 75% and 100%, respectively). Plants expressing OMMV *p6* showed a slight lower DSI at 10 dpi (60%), but also reached 100% at 16 dpi, showing only a delay in the appearance of symptoms. At the same time points, plants expressing OMMV *CP* presented considerably lower DSIs (32.5% and 57.5%), indicating a tolerance to OMMV infection. In contrast, plants expressing both OMMV CP and p6 showed a maximum DSI of 20% at 16 dpi, indicating a very high tolerance to OMMV infection. Additionally, in the experiments, an average of 60% of the plants expressing OMMV *CP* and *p6* did not present any viral symptoms at 16 dpi, suggesting a resistance to OMMV. Plants expressing OMMV *CP* also showed some resistance, although in a lower level (20%). Plants were monitored until 30 dpi, and the highest DSI was 60% in plants expressing OMMV *CP*, and 30% in plants expressing both OMMV *CP* and *p6*, no plants reaching the maximum disease index.

**Figure 4:**
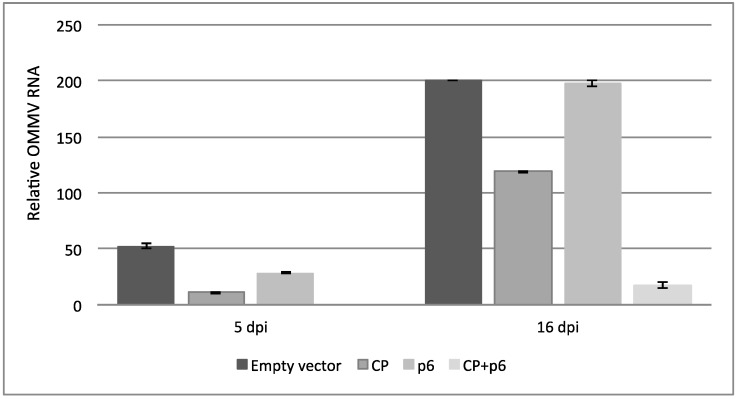
Viral accumulation levels in upper non-inoculated leaves of three biological replicates, at five and 16 days after OMMV inoculation.

Viral accumulation levels were determined by real time RT-qPCR (Figure 4), and the results are consistent with the disease severity symptoms observed. At five dpi, the mean viral accumulation ± SE in the upper non-inoculated leaves of plants expressing OMMV*CP* + *p6* was 0.0, 11.0 ± 2 in OMMV*CP*, 28.5 ± 0.5 in OMMV*p6*, and 52.5 ± 2.5 in plants expressing the empty vector. Individual pairwise comparisons revealed significant differences (*p* < 0.01) between all of the treatments. At 16 dpi, the mean viral accumulation ± SE in the upper non-inoculated leaves of plants expressing OMMV*CP* + *p6* was 17.5 ± 2.5, 119.0 ± 1.0 in OMMV*CP*, 197.5 ± 2.5 in OMMV*p6*, and 200.0 ± 0.0 in plants expressing the empty vector. Individual pairwise comparisons revealed significant differences between empty vector versus OMMV*CP* (*p* < 0.008); empty vector versus OMMV*CP* + *p6* (*p* < 0.0032); OMMV*CP* versus OMMV*p6* (*p* < 0.0011); OMMV*CP* versus OMMV*CP* + *p6* (*p* < 0.005), and OMMV*CP* + *p6* versus OMMV*p6* (*p* < 0.0029). No significant differences were found between empty vector and OMMV*p6* (*p* > 0.4205).

Both visual observations and quantitative RT-PCR indicate that OMMV *CP*, and remarkably OMMV *CP* together with *p6*, attenuate viral symptoms and reduce viral accumulation levels.

## 4. Discussion

Most plant viruses have evolved by encoding one or more silencing suppressors as a counter-response to the antiviral plant gene silencing defense mechanism [9]. Members of the *Tombusviridae* family share common features, but show different strategies to suppress silencing. Tombusviruses p19 protein, the equivalent p14 protein of the aureusviruses, the replicase proteins of the dianthoviruses, and the CP of the carmoviruses (alpha and beta) have been identified as Tombusviridae silencing suppressors.

Members of the *Tombusviridae* family present a high diversity of viral suppressors. The closest related genera, the carmoviruses, use the *CP* as silencing suppressor. However, in opposition to the carmoviruses CP, the CP of necroviruses lacks a protruding domain, and has low similarity to the *CP* of carmoviruses. In addition, the genome of OMMV does not present proteins equivalent to tombusviruses p19 and aureusviruses p14, and the necroviruses replicase has a very low similarity to the replicase of the dianthoviruses [36].

In this study, the complete OMMV genome was screened for the presence of a gene with potential RNA silencing suppression activity. Co-infiltration assays using the *N. benthamiana* 16C line, *GFP*-transformed, revealed that the full genome of OMMV encodes RNA silencing suppressors with activity similar to the strong suppressor Tav-*2b*. When each of the individual proteins were tested separately, *CP* and *p6* were capable of inhibiting ds*GFP*-induced local silencing, at different levels. However, this was lower than when the entire genome was used, as seen by enhanced GFP fluorescence and *GFP* mRNA levels. Despite no experiments having been done using a combination of *p6* and *CP*, the other ORFs not showing a suppressor activity seems to suggest that OMMV silencing suppression-enhanced activity is due to a coordinate and complementary action of both *CP* and *p6* as silencing suppressors. The analysis of each protein separately demonstrated that most suppressor activity is due to the *CP*, with *p6* showing a much lower inhibition of local RNA silencing.

This finding is in line with previous data on OMMV mutants containing several changes in *CP* sequence, which exhibited different levels of symptoms in indicator plants, suggesting a clear role of that protein in symptom modulation [24].

The combination of several functions in the same protein may be advantageous for the virus.

This is the first time that two independent proteins were found to act as silencing suppressors in a member of the *Tombusviridae* family similarly to that shown in some members of *Closteroviridae* [37] and *Filoviridae* [38].

The existence of different viral suppressor proteins in one virus seems to be an advantage in targeting different pathways of host defense at the cellular level as it allows for a more successful host infection. Viruses with single suppressors may only gain this advantage in mixed infections with others that possess distinct silencing suppressors with different targeting modes, thus causing a more effective interference with the plant defense mechanism that results in increased symptoms, higher viral accumulation, and favoring cell-to-cell movement [39,40,41]. This has been observed with PVX and CMV that encode distinct suppressors that target both intracellular and intercellular silencing, leading to enhanced viral accumulation and symptoms characteristic of synergistic effect, in double infections [42].

Synergistic effects have been seen between OMMV and the close related OLV-1 during co-infection. OMMV is only acquired from the soil when OLV-1 is present, in which case both viruses are able to invade the plant and spread throughout the plant, causing systemic symptoms instead of the typical local lesions induced by each virus separately [43]. In fact, it is interesting to notice that both OMMV silencing suppressors are predicted to be involved in movement: p6 in cell-to-cell movement, and CP in systemic movement [44].

The discovery of OMMV viral suppressors have prompted us to explore their use in the development of OMMV-resistant plants through pathogen-derived resistance (PDR) based on RNA silencing.

The development of viral resistant plants has been a matter of study for many years and different levels of resistance have been obtained varying with the type of molecules and viral genome region used to trigger RNA silencing [45,46,47]. As an alternative to transgenic plants and to overcome their associated biosafety and legislative constrains, transient RNA silencing systems have been developed consisting on the direct delivering of different RNA silencing molecules into plants [48,49,50].

The expression of self-complementary hairpin-RNA, in opposition to single-sense or antisense constructs, induces a high level of RNA silencing in plants due to the production of dsRNA through the transcription of the hairpin structure; in addition, the presence of an intron in between the complementary regions stabilizes the construct and enhances silencing efficiency [51,52,53].

Tests were conducted to find out whether the expression of artificial RNAs targeting the suppressors *CP* and *p6* could counteract the suppressor function and confer resistance to OMMV using hairpin constructs. Two hairpin constructs targeting the *p6* and the *CP* were agroinfiltrated in plants to transiently express long pathogen-related dsRNAs and pre-activate plant RNA silencing machinery through the procession of dsRNAs into siRNAs that are responsible for the degradation of invading viral RNA.

For the construction of the *p6* hairpin, the full *p6* was used; for the *CP* hairpin construct, the first 141 nt of the *CP* were removed (OMMV *CP*minus141) in order to reduce the potential biosafety risks associated with the production of CP molecules, as well as exclude CP-mediated resistance events; still, a long fragment was used so that a higher number of potentially active virus-specific siRNAs would be present and specifically target the coding region of *CP*. 

This study shows that the expression of OMMV *CP*minus141 and OMMV *p6* interfered with the multiplication of OMMV, as the plants expressing them resulted in a highly substantial reduction in viral accumulation and symptoms attenuation, proving their effectiveness against OMMV infection. The percentage of resistant plants obtained in this study (60%) is similar to the highest percentages of viral resistance obtained in other studies [50,53,54].

Plants transiently expressing OMMV *CP*minus141 interfered with the multiplication of OMMV; however, it was not as effective against OMMV infection as plants expressing both that and OMMV *p6*, as just 20% of the plants were OMMV-resistant. No resistant plants were obtained when OMMV *p6* was being expressed; however, a slight delay in the appearance of symptoms occurred when compared to the control. These results show that OMMV can counteract these mechanisms by inhibiting silencing. Only when plants expressed both OMMV suppressors, *CP* and *p6*, was a high efficient RNA silencing mechanism triggered. This has also been observed for CTV, where resistance is only achieved when the three viral silencing suppressors were targeted simultaneously [55].

The findings obtained here contribute to the knowledge on resistance induction frequencies, and are of great importance for the future development of OMMV-resistant plants that are either transgenic or, as an alternative, in transient systems by directly delivering RNA-silencing molecules into plants, as to overcome the lack of a regulatory framework and the strong opposition associated with transgenic plants. Additionally, resistance to other closely related viruses may be achieved, as viruses with less than 15–20% difference at the sequence level may confer protection to each other [56,57]. Such small differences exist between OMMV and other necroviruses such as TNV-D and OLV-1, whose co-infections are frequent in nature [58]. It is common for plants to be invaded by several viruses, and this study shows the most efficient OMMV transgenes that confer resistance to OMMV, which may be used in multiple combined sequences assembled with other viruses that are expected to infect the crop to achieve protection and a broader resistance against a wide range of viruses.

Besides helping understandings of the interaction between viruses and hosts, the knowledge on viral suppressors may also have other commercial impacts, as in the production of heterologous proteins using bioreactor plants. Host RNA silencing has shown to reduce the efficiency of gene expression in plants [59]. In this way, the co-expression of a silencing suppressor and the target gene may be an attractive option to reduce problems in transgene expression. This is essential to respond to the continuous increase in the demand to produce large amounts of recombinant proteins for the industry, for which plants are one of the most effective and safe systems for large-scale production.

## Figures and Tables

**Table 1:** Primers used in the Gateway recombination reactions. Recombination sequences specific for the gateway system are underlined. OMMV: *Olive mild mosaic virus*.

Primer Name	Primer (5′–3′)	Region Amplified	Location in OMMV Genome	Fragment Length (bp)
attB1	GGGGACAAGTTTGTACAAAAAAGCAGGCT	-	-	-
attB2	GGGGACCACTTTGTACAAGAAAGCTGGGT			
OMMVattB1	AAAAAGCAGGCTAGTATACATACCAAGTATA	OMMV	1–19	3707
OMMVattB2	AGAAAGCTGGGTGGGGTCGGGCAAAGGCC		3667–3683	
OMMVp23attB1	AAAAAGCAGGCTAGGATAAAATGGAGCTCAC	*p23*	52–70	642
OMMVp23attB2	AGAAAGCTGGGTCCTATTTGGCCCGGAAGGCC		650–669	
OMMVP82attB1	AAAAAGCAGGCTAGGATAAAATGGAGCTCAC	*p82*	52–70	2208
OMMVP82attB2	AGAAAGCTGGGTATCAGTTTGGTAATCCATTG		2216–2235	
OMMVp8attB1	AAAAAGCAGGCTTTTAATCAATGGATTACCA	*p8*	2210–2228	267
OMMVp8attB2	AGAAAGCTGGGTACACAGCCATAACTCAAAAG		2433–2452	
OMMVp6attB1	AAAAAGCAGGCTCTTTTGAGTTATGGCTGTGT	*p6*	2433–2452	208
OMMVp6attB2	AGAAAGCTGGGTTGTCTATTTTGCGATCG		2600–2616	
OMMVCPattB1	AAAAAGCAGGCTACCAAAACATGCCTAAGAG	*CP*	2628–2646	842
OMMVCPattB2	AGAAAGCTGGGTTCAAACGTTAATGGTAGGG		3427–3445	

**Table 2:** Primers used in RT-qPCR. GFP: green fluorescent protein.

Gene	Primer Name	Primer (5′–3′)	Reference
*Protein phosphatase 2A*	PP2ArtFW	GACCCTGATGTTGATGTTCGCT	[30]
	PP2ArtREV	GAGGGATTTGAAGAGAGATTTC	
*F-box protein*	FBOXrtFW	GGCACTCACAAACGTCTATTTC	[30]
	FBOXrtREV	ACCTGGGAGGCATCCTGCTTAT	
*GFP*	GFP-ER Taq-F	GCCAACACTTGTCACTACTTTCTC	[28]
	GFP-ER Taq-R	GTAGTTCCCGTCGTCCTTGAAG	
*OMMV p23*	OMMVP23rtFW	CGAGTCCGCAAGCAGAAGAAG	This study
	OMMVP23rtREV	GGGTAGACCAAACTCGGCA	
*OMMV p82*	OMMVP82rtFW	TCCAAGACGCCCCGAAAC	This study
	OMMVP82rtREV	TGGTTACAGGGGAATGACGC	
*OMMV p8*	OMMVP8rtFW	GCTCAGAAATCGCAGCAAGG	This study
	OMMVP8rtREV	TGTCACGGTAATGGTCTGTTCT	
*OMMV p6*	OMMVP6rtFW	TGTGTCGCTGCTGTGATACTT	This study
	OMMVP6rtREV	TTGCAAGGATGAGGATGAGAAT	
*OMMV CP*	OMMVCPrtFW	TGTCCAGCCACAGCTCTCAT	This study
	OMMVCPrtREV	TTCGATGAACTCAATCTCATATCGC	

**Table 3:** Details of the two-factor permutational analysis of variance (PERMANOVA) pairwise tests with open reading frames (“ORFs”) (eight levels, fixed) and “Time” (three levels, fixed) for all of the analyzed variables. Bold values highlight significant effects (*p* < 0.05).

Pairwise Tests	3 dpi	5 dpi	10 dpi
ORFs			
*GFP* vs. Tav-*2b*	**0.0001**	**0.0001**	**0.0001**
*GFP* vs. OMMV	**0.0001**	**0.0001**	**0.0001**
*GFP* vs. *p23*	0.9153	**0.0001**	**0.0001**
*GFP* vs. *p82*	0.6178	**0.0001**	0.3574
*GFP* vs. *p8*	**0.0002**	**0.0001**	**0.0001**
*GFP* vs. *p6*	**0.0001**	**0.0001**	**0.0001**
*GFP* vs. *CP*	**0.0001**	**0.0001**	**0.0001**
Tav-*2b* vs. OMMV	**0.0129**	0.0518	**0.0001**
Tav-*2b* vs. *p23*	**0.0001**	**0.0001**	**0.0001**
Tav-*2b* vs. *p82*	**0.0001**	**0.0001**	**0.0001**
Tav-*2b* vs. *p8*	**0.0001**	**0.0001**	**0.0001**
Tav-*2b* vs. *p6*	**0.0001**	**0.0001**	**0.0001**
Tav-*2b* vs. *CP*	**0.0001**	**0.0004**	0.8368
OMMV vs. *p23*	**0.0001**	**0.0001**	**0.0001**
OMMV vs. *p82*	**0.0001**	**0.0001**	**0.0001**
OMMV vs. *p8*	**0.0001**	**0.0001**	**0.0001**
OMMV vs. *p6*	**0.0001**	**0.0002**	**0.0001**
OMMV vs. *CP*	**0.0001**	**0.0134**	**0.0001**
*p23* vs. *p82*	0.6137	**0.0016**	0.6807
*p23* vs. *p8*	**0.0001**	**0.0017**	0.6569
*p23* vs. *p6*	**0.0001**	**0.0001**	**0.0001**
*p23* vs. *CP*	**0.0001**	**0.0001**	**0.0001**
*p82* vs. *p8*	**0.0001**	0.9977	0.6799
*p82* vs. *p6*	**0.0001**	**0.0001**	**0.0001**
*GFP* vs. Tav-*2b*	**0.0001**	**0.0001**	**0.0001**
*GFP* vs. OMMV	**0.0001**	**0.0001**	**0.0001**
*GFP* vs. *p23*	**0.0001**	**0.0001**	**0.0001**
*GFP* vs. *p82*	**0.0001**	**0.0001**	**0.0001**
